# Polymer Tablet Matrix Systems for the Controlled Release of Dry *Betula pendula* Leaf Extract

**DOI:** 10.3390/polym15173558

**Published:** 2023-08-26

**Authors:** Dimitar Penkov, Paolina Lukova, Hristo Manev, Stela Dimitrova, Margarita Kassarova

**Affiliations:** 1 Department of Pharmaceutical Sciences, Faculty of Pharmacy, Medical University of Plovdiv, 4002 Plovidv, Bulgaria; 2 Department of Pharmacognosy and Pharmaceutical Chemistry, Faculty of Pharmacy, Medical University of Plovdiv, 4002 Plovdiv, Bulgaria; 3 Department of Medical Physics and Biophysics, Faculty of Pharmacy, Medical University of Plovdiv, 15A Vasil Aprilov Blvd, 4002 Plovdiv, Bulgaria; 4 Department of Bioorganic Chemistry, Faculty of Pharmacy, Medical University of Plovdiv, 15A Vasil Aprilov Blvd, 4002 Plovdiv, Bulgaria; 5 Research Institute at Medical University of Plovdiv, 4002 Plovdiv, Bulgaria

**Keywords:** polymer tablets, sustained release, plant extract, ethylcellulose, hydroxypropyl methylcellulose

## Abstract

The aim of the study was to develop polymer matrix tablets with modified release of dry *Betula pendula* leaf extract and to investigate basic parameters influencing the drug release pattern. To fully assess the statistical significance of the influence of the individual factors in the tablet formulation development as well as the combination of them, Tukey’s tests and a complete 3^3^ factor analysis of variance (ANOVA) were applied. The following three factors were studied at three levels (low, medium and high): influence of the hydrophobic/hydrophilic polymer ratio Ethylcellulose (EC)/Hydroxypropyl methylcellulose (HPMC) (40/60, 25/75 and 10/90), influence of HPMC molecular weight (500 kDa, 750 kDa and 1150 kDa), and influence of the compression force applied (1 t, 1.5 t and 2 t). The effect of these varied parameters on the drug release parameter t80 was evaluated statistically. Twenty-seven tablet models were formulated, including all possible combinations of the variables. The obtained drug release profiles demonstrated that a 25/75 (EC/HPMC) ratio was the most suitable for prolonging the release process. Increasing the molecular weight of HMPC from 500 kDa to 750–1150 kDa and applying higher compression force significantly influenced the studied t80 values and caused sustained drug release (t80 up to 7.97 h). The combination of the hydrophilic HPMC polymer with the hydrophobic EC can result in the formation of a promising drug-carrying matrix, offering effective control of the drug release process.

## 1. Introduction

Modern phytotherapy is a successful combination of traditional knowledge about the therapeutic use of medicinal plants and novel pharmacological, pharmacognostic and technological approaches based on an in-depth knowledge of the herbal chemical composition, mechanisms of action of the biologically active substances, and formulating the most appropriate pharmaceutical form [1]. The main purpose of most pharmaceutical forms is to deliver the active substances to a desired site in the body and to maintain their therapeutic concentration over a specific period of time. The conventional pharmaceutical forms are often associated with the need for frequent and repeated administration, which can be a serious disadvantage. Plant extracts usually have good solubility as well as rapid biotransformation, and providing an adequate dose over a sufficiently long period of time can be essential for the therapeutic effect of the administered formulation [2,3].

Betulaceae is a large family containing more than 150 species of trees and shrubs, primarily distributed in the Northern Hemisphere [4,5]. Products containing extracts from different parts of silver birch (*Betula pendula* Roth, Betulaceae) are known and used in traditional medicine and phytotherapy in various forms [6,7]. In our previous studies, we formulated a dry, standardized birch leaf extract and studied a number of its biological effects (such as antitumor, antimicrobial, antispasmodic, antioxidant, etc.) [8,9]. Despite the promising biological activity in many of these studies, the ability to maintain a high plasma concentration over a prolonged period of time has emerged as a major drawback. One possibility to overcome this problem was the inclusion of the extract in a modified release oral tablet formulation. Oral administration of drugs is the most common route for drug administration because it is non-invasive, economically accessible and well accepted by patients. Furthermore, tablet dosage forms as solid preparations can provide enhanced drug stability, especially for biologically active substances easily susceptible to degradation, and with the appropriate selection of excipients, a precise regulation of the drug release rate can be achieved.

Modified-release pharmaceutical forms are widely used due to the numerous advantages they offer. The use of different types of polymers to control the drug release process represents one of the main aspects of the development of different types of matrix systems. Hydrophilic matrices are often the first choice in the development of modified-release pharmaceutical forms [10,11,12]. They are usually monolithic systems containing one or more hydrophilic polymers [13]. Our preliminary work showed that, in this case, a hydrophilic polymer alone is not sufficient to provide sustained release. For this reason, model compositions containing a combination of hydrophilic and hydrophobic polymers were developed. It has been reported that by combining a hydrophilic HPMC matrix with a small amount of ethylcellulose, the release of highly soluble drugs can be delayed and the release process can be better controlled [14]. Adjusting the ratio of hydrophobic and hydrophilic polymers offers additional control options. Although the production process of the polymer matrix system is relatively simple, a great challenge is the detailed explanation of the release mechanism of the included active substances. Some of the factors that have the most significant influence on the processes of diffusion, erosion and drug release are the concentration and ratio of the polymers used, their molecular weight and specific technological parameters in the production process.

An increasing number of reports on the use of experiments in the development and optimization of various drug delivery systems can be found in the literature. Different statistical design approaches are often used as a powerful tool to optimize process parameters and pharmaceutical form characteristics [15,16,17,18,19]. A full factorial design is a systematic design that allows a full assessment of the influence of several factors (independent variables) and their interactions on a single (dependent) variable. Such models are often applied to understand the interactions between factors (composition, excipient concentration, parameter values, etc.) and the desired characteristics of the tablets [20,21,22].

Therefore, the aim of the research was to design polymer tablet matrix systems as a platform providing delayed release of the included dry *Betula pendula* leaf extract and offering improved biopharmaceutical characteristics. The work is focused on the precise optimization of various factors such as compression parameters, type, concentration and molecular weight of the polymers used, which affect the characteristics of the formulation and the drug release process. The influence of the varied parameters, both individually and in combinations, was thoroughly evaluated using statistical DOE (design of experiments) approaches.

## 2. Materials and Methods

### 2.1. Materials

Hyperoside (analytical standard), ethylcellulose (48.0–49.5% (*w/w*) ethoxyl basis), magnesium stearate and talc (technical grade) were purchased from Sigma Aldrich (Sigma-Aldrich Chemie GmbH, Taufkirchen, Germany). Hydroxypropyl methylcellulose of different molecular weights (Methocel K100M, Methocel K15M and Methocel K4M (hydroxypropyl methylcellulose) was purchased from The Dow Chemical Company (Midland, MI, USA). Other chemicals and reagents used were of analytical grade. Birch leaves (*Betula pendula*, Roth) were collected from the Rhodopes Floristic Region of Bulgaria in June–August 2019. The leaves were identified by their macroscopic and microscopic characteristics according to the requirements of the European Pharmacopoeia 9. They were dried in the shade without using high temperatures to preserve biologically active substances. Dried leaves were ground to 5 mm in size.

### 2.2. Preparation of Dry Betula pendula Leaf Extract

Dry *Betula pendula* leaf extract was prepared using the Mini Spray Dryer B-290, Buchi (BÜCHI Labortechnik, Flawil, Switzerland). Ethanol extracts and dispersed colloidal silica were homogenized prior to and throughout the spray drying process by stirring with an electromagnetic stirrer at 300 rpm. Solutions were fed to a 0.7 mm spray nozzle. Nitrogen pressure was kept constant during the process—6 bar at a gas flow rate of 600 L/h, a peristaltic pump speed of 15%, an inlet temperature of 140 °C and aspiration of 95%.

### 2.3. Experimental Design

The development of the model tablet formulations containing dry extract was performed using 3^3^ full factorial designs. Tablets were prepared by the wet granulation method with an average weight of 0.700 g. Different concentrations and ratios of the hydrophobic polymer ethylcellulose (EC) and the hydrophilic polymer hydroxypropyl methylcellulose (HPMC) (EC/HPMC 40/60, 25/75 and 10/90, respectively) were used, varying the viscosity of the HPMC used (K4M—500 kDa, K15M—750 kDa and K100M—1150 kDa) and the applied compression force (1 t, 1.5 t and 2 t) (Table 1 and Table 2).

To fully assess the statistical significance of the influence of the individual factors as well as the combination of them, a complete 3^3^ factor analysis of variance (ANOVA) was applied. Three factors (independent variables) were determined and studied in experimental design at three levels: low (−1), medium (0) and high (+1), as follows: influence of the hydrophobic/hydrophilic polymer ratio (EC/HPMC), influence of HPMC molecular weight, and influence of the compression force applied, and their effect was evaluated on the dependent variable t80, indicating the time required to release 80% of the extract included in the tablet. Table 3 shows the 27 tablet models developed (from F1 to F27), which include all possible combinations of variables. All measurements were performed three times, and the results were presented as mean values with their statistical deviations.

### 2.4. Granulation and Tableting Process

The required amounts of ethylcellulose, hydroxypropyl methylcellulose and dry extract were mixed, ground and sieved through a 0.5 mm sieve. The wet mixing was carried out with the gradual addition of 96% ethanol and continuous homogenization. Granulation was performed on a granulator ERWEKA AR 403 FGS (ERWEKA GmbH, Langen, Germany) through a 2 mm sieve. The wet granular mass was dried at 45 °C to a moisture content of 3.5%. The classification of the granules was performed through a 1.6 mm sieve. The granular mass was dusted with the required amounts of talc and magnesium stearate for 5 min. The tablet compression was performed on a single-punch eccentric tablet press, the ERWEKA EP-1 with 13 mm punches at three different compression forces.

### 2.5. Characterization of Granules and Tablets

The rheological characteristics of the 9 models of granular mixtures obtained were determined according to the pharmacopoeial methods for evaluation: angle of repose, flow rate through an orifice, compressibility index (Carr’s index) and Hausner ratio. The control parameters of the prepared 27 tablet models were determined according to the tests described in the European Pharmacopoeia 9, respectively: uniformity of mass, disintegration time, resistance to crushing and friability. Analyses were performed with laboratory equipment: tapped density tester SVM Erweka, disintegration tester Erweka—ZT 121, tablet hardness tester Erweka—TBH 125 and friabilator Erweka—TA 120.

### 2.6. In Vitro Drug Release

Dissolution tester Sotax CH 4123, Apparatus 2 (paddle), was used at the following operating parameters: test medium: 900 mL (0.1 N HCl buffer solution of pH 1.2 as Simulated Gastric Fluid (SGF), temperature 37 ± 0.5 °C, speed 150 rpm). The release of the incorporated plant extract from the polymer systems was evaluated by quantitative analysis of the flavanoid hyperoside using high-performance liquid chromatography (HPLC). HPLC system Varian Prostar with a column Hitachi C18 AQ (250 nm × 4.6 mm, 5 µm) and PDA detector was used. A mobile phase A (H_2_O, pH 3.7): B (CH_3_CN) was used in a gradient mode from 90(A):10(B) to 10(A):90(B) with a flow rate of 0.9 mL/min. Detection was performed at 335 nm. The quantitative determination was performed by the method of the external standard. Results were presented as mean ± SD values, provided from 3 independent measurements. The drug release profile was monitored for a period of 8 h. The dissolution profiles of all tested models were fitted to zero-order, first-order, Higuchi and Korsmeyer–Peppas kinetic models.

## 3. Results and Discussion

### 3.1. Rheological Characteristics of the Granules and Control Parameters of the Obtained Tablets

The results from the rheological tests of the model granules obtained after wet granulation are presented in Table 4.

The results of the rheological tests performed showed that all tested models met the pharmacopoeial requirements and had acceptable rheological properties to perform the tablet compression process [23]. Regarding the Hausner factor, all tested models showed results below 1.25, which is an indicator of free flow. The Carr index for all tested models except G5 and G8 indicated good compressibility. All models except G5 showed an angle of repose below 25°, which is an indicator of excellent flowing properties.

From the nine granular models presented, 27 tablet models were obtained using three different compression forces (1 t, 1.5 t and 2 t). The varied parameters of the 27 model tablet compositions—uniformity of mass, disintegration time, friability and hardness—are presented in Table 5.

The model tablets showed good uniformity of mass and relatively high hardness, varying in the range from 88 N to 165 N (which correlated with the applied compression force). All tested tablets showed very low friability, ranging from 0.01% to 0.126%.

### 3.2. In Vitro Drug Release and Dissolution Profiles

Our preliminary investigations have shown that a satisfactory delay in the release process from tablet models containing only hydrophilic polymers (HPMC) could not be achieved. The combination of hydrophobic ethylcellulose with the hydrophilic hydroxypropyl methylcellulose, as well as the change in the hydrophobic-hydrophilic polymer ratio, offers ample opportunities to control the release process. There is also evidence in the literature that combining a hydrophilic matrix of HPMC with a small amount of ethylcellulose results in a delay in the initial intensive release of well-soluble drugs and more effective control of the release process [14].

Usually, the presence of a highly soluble substance in a matrix of HPMC generates an additional osmotic gradient, resulting in a faster rate of polymer swelling and a large increase in gel thickness. In the presence of water, the mobility of the polymer chains is enhanced, resulting in the gradual transformation of a glassy matrix into a rubbery, swollen gel. At higher polymer concentrations, the viscosity of the gel matrix is increased, which results in a decrease in the effective diffusion coefficient of the drug [24]. In a study by Enayatifard et al., the authors try to develop an oral controlled matrix drug delivery system for a highly water-soluble drug (similar to dry birch leaf extract) using hydrophilic (HPMC) and hydrophobic (EC) polymers either alone or as a blend. Models containing only the water-soluble polymer HPMC demonstrate the expected trend, where the release rate decreased as the concentration of HPMC increased. The authors found that the inclusion of different concentrations of EC (alone) affected the release process but was not efficient enough and had a highly pronounced burst effect. The delay in the release process is usually attributed to the decreased penetration of the solvent molecules in the presence of the hydrophobic polymer, leading to reduced diffusion of the drug from the matrix. It is important to note that the concentration of the hydrophobic polymer is of utmost importance, and the increased amount does not necessarily slow down the release process. This is related to the penetration theory, according to which drug release occurs by dissolution of the active ingredient through capillaries composed of interconnecting drug particle clusters and the pore network. Models containing a combination of both polymers with a predominant content of HPMC demonstrate effective control of the release process over a period of 8 h and a missing initial burst effect [25]. Similar trends were found in the study of the release process of our tablet models.

**Figure 1:**
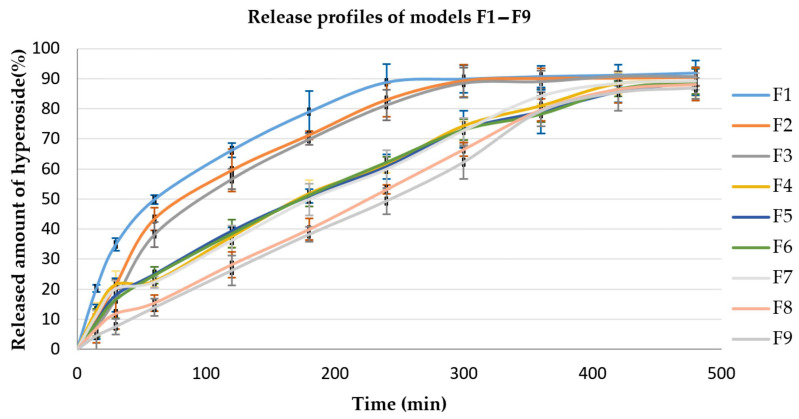
Hyperoside release profiles from models F1–F9, EC/HPMC ratio 40/60 (*n* = 3).

**Figure 2:**
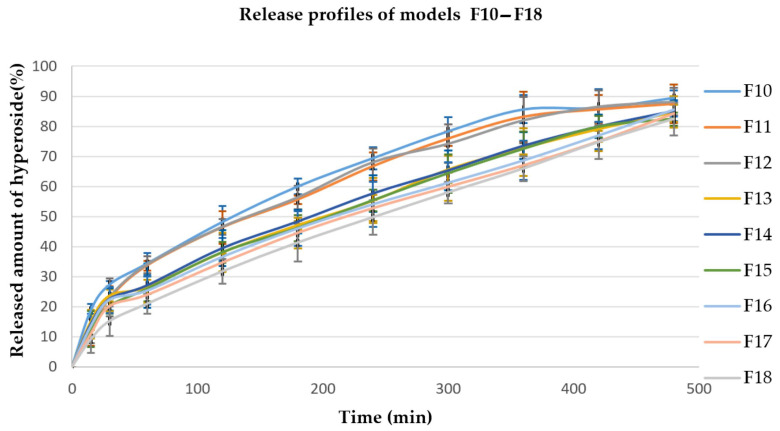
Hyperoside release profiles from models F10–F18, EC/HPMC ratio 25/75 (*n* = 3).

**Figure 3:**
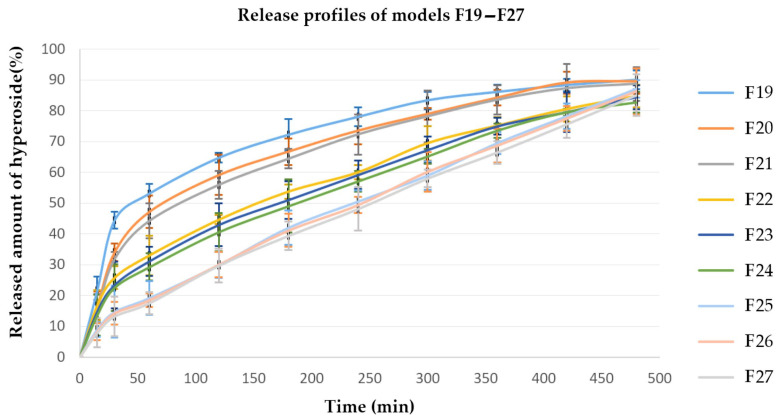
Hyperoside release profiles from models F19–F27, EC/HPMC ratio 10/90 (*n* = 3).

Other factors that could significantly affect the release process are the viscosity of the polymer used as well as the compression force applied in the preparation of the tablets. Figure 1, Figure 2 and Figure 3 present the hyperoside release profiles of the developed tablet models.

To determine the release kinetic mechanism, in vitro drug release data were fitted to different kinetic models: zero-order, first-order, Higuchi and Korsmeyer–Peppas. The R^2^ values obtained from different mathematical models were evaluated and presented in Table 6. The table also presents data on the diffusion exponent n, considered an indicator of the transport mechanism through the polymer matrix [26,27].

Korsmeyer–Peppas was determined to be the most suitable kinetic model, describing the process of in vitro drug release from all tested tablet models and showing the highest values of R^2^ (from 0.992 to 0.999). The Korsmeyer–Peppas mathematical model is often used to analyze the release of drugs from polymer systems and reflects the linear relationship between the logarithmic amount of substance released over time t (Log M_t_/M_0_ and Logt).

In most of the studied models (except models F1, F19, F20 and F21), the established values for the diffusion exponent n were in the range of 0.45 to 0.95. These results confirmed the assumption that the mechanisms of diffusion and erosion were involved simultaneously and influenced the process of releasing the substance included in the matrix system. Values of n less than or equal to 0.45 are an indicator for a Fickian diffusion transport mechanism (Case I transport). Values of n between 0.45 and 0.95 usually indicate non-Fickian diffusion (anomalous transport), which includes a combination of diffusion and erosion in the release mechanism. Values above 0.95 indicate a dominant erosion release mechanism (Case II transport and Super Case II transport). The relative complexity of the matrix system containing a combination of hydrophobic and hydrophilic polymers is a prerequisite for the release mechanism to be influenced by more than one process [27,28,29].

In a study investigating the influence of HPMC with different molecular weights in composite matrix tablets, authors found values for the diffusion exponent n ranging from 0.48 to 0.50, indicating that the release was governed by anomalous transport, i.e., the combination of the drug diffusion through the gel layer and the erosion of the polymer gel layer [30].

### 3.3. Influence of the Hydrophobic/Hydrophilic Polymer Ratio (EC/HPMC)—Factor A on t80

To assess the effect of the EC concentration on the release process, model formulations were developed by varying the ratio between the hydrophobic and hydrophilic polymers (10%, 25% and 40% EC content). When comparing analogous models containing HPMC with the same molecular weight and obtained with the same compression force, the following trend is clearly observed: increasing the amount of the hydrophobic polymer from 10% (level −1) to 25% (level 0) leads to significant elongation of t80 (Table 7). Model F19 containing HPMC with a molecular weight of 500 kDa, 1 t pressure and an EC content of 10% shows a t80 of 4.40 h, while model F10 obtained under the same conditions but containing 25% EC shows a t80 of 5.26 h. A similar comparison can be made between the models F22 and F13 and F25 and F16, which contain medium molecular weight (750 kDa) and high molecular weight (1150 kDa) HPMC, respectively. Although less pronounced, the differences follow the same trend. Model F22 containing 10% EC shows a t80 of 7.01 h, and its similar model F13 containing 25% EC shows a t80 of 7.25 h.

The release profiles of the experimental models showed that increasing the EC concentration led to a reduced initial release and prolongation of t80. This is probably due to the fact that the presence of the hydrophobic polymer in the matrix slows down the intensive water ingress in the initial moments, even before the formation of a viscous hydrogel, hence the dissolution of the included plant extract and effective control of the release process. It is noteworthy that more significant differences are observed in models containing low-molecular-weight HPMC. This was expected, as the high molecular weights of K15M and K100M create a more viscous hydrogel layer, which slows down the release process more efficiently. However, as the concentration of EC increased, the observed trend changed. Comparison of the models F10 (containing 25% EC, low molecular weight HPMC and obtained at 1 t pressure) and F1 (containing 40% EC, low molecular weight HPMC and 1 t pressure) showed a decrease in t80 from 5.26 h to 3.12 h. This pattern was preserved in other analogous models containing medium- and high-molecular-weight HPMC and 25% and 40% ethylcellulose, respectively. In each case, models containing 40% EC showed faster release of the included extract and correspondingly lower values for t80. This can be explained by the fact that the large hydrophobic ethylcellulose molecules create local disturbances in the integrity of the viscous hydrogel layer, leading to lower retention of the soluble substances and faster drug release.

A similar pattern has been reported by other authors. Madgulkar et al. use response surface methodology for the formulation and optimization of sustained-release tablets of venlafaxine resinates. HPMC and EC were taken as independent variables in the central composite design for two factors at three levels. In vitro dissolution studies were performed to study the release kinetic parameters. In this case also, like in other matrix systems of similar type, the diffusion exponent n values are indicative of a coupling of diffusion and erosion mechanisms. Increased n values are observed with an increase in HPMC content, even at higher EC levels. The drug released was measured at the 2nd and 8th hours. A general tendency is clearly noticed when comparing the presented models: as the amount of HPMC increases, the release process slows down. However, the amount of EC does not affect the release process in the same way. Comparing the models F1–F3 containing the lowest concentration of HPMC (41.62 mg) and increasing concentrations of EC (41.62, 83.24 and 124.86, respectively) shows that the double increase in the amount of EC (model F1 to model F2) leads to a delay in the release process and a correspondingly smaller amount of released drug at the 2nd and 8th hours. However, when the concentration is further increased (model F3), the release process does not slow down further, and even a greater amount of released substance is observed compared to model F2. In the models with increased HMPC concentration, the tendency is maintained—low and medium EC values lead to slower release, compared to the highest concentrations, which generally show faster release of the included drug substance [28].

The results of Tukey’s test on the effect of the hydrophobic/hydrophilic polymer ratio (EC/HPMC) on the release time (t80) are presented in Table 8. Data analysis showed that each of the differences in the mean values was statistically significant, i.e., the probability of making a type I error was less than the permissible significance level of 5%. EC/HPMC ratio of 10/90 demonstrated a 0.3148 lower value for t80 compared to 25/75. The 25/75 ratio showed a 0.3148 higher value for t80 compared to 10/90 and 1.6233 higher compared to the 40/60 ratio, respectively.

It can be concluded that the addition of EC to the hydrogel matrix improves the control of the release process, but only up to a certain polymer concentration. Furthermore, increasing concentration has the opposite effect and rather accelerates the release of the drug substances.

### 3.4. Influence of the HPMC Molecular Weight—Factor B on t80

The molecular weight of the hydrophilic polymer used is essential for the rate and extent of drug release. When comparing analogous models obtained with the same hydrophobic/hydrophilic polymer ratio of 40/60 and the same compression force of 1 t (e.g., models F1, F4 and F7), the following trend is clearly outlined: increasing the molecular weight of the hydrophilic polymer used (increase of factor B from level −1 to level 0, as well as from level 0 to level +1) leads to a delay in the release of the included plant extract. This difference is more significant when comparing the models prepared with the lowest viscosity polymer, K4M, and those with medium viscosity, K15M (models F1 and F4). When comparing the F4 and F7 models (including the medium molecular weight K15M and the high molecular weight K100M), the trend was preserved, but although statistically significant, it is much less pronounced.

In a study from 2014, Jain and co-authors investigated the effect of HPMC molecular weight and concentration on the in vivo erosion behavior of HPMC matrix tablets. Different formulations were investigated, containing varying proportions of two HPMC grades with different molecular weights. The in vivo erosion behavior and gastrointestinal transit were investigated using magnetic marker monitoring. The authors concluded that the erosion is strongly dependent on the composition of the formulations. Models containing a larger amount of high molecular weight HPMC or a higher content of HPMC exhibit a relatively slower erosion rate, and vice versa. Therefore, the appropriate erosion characteristics of HPMC matrix tablets can be modulated by manipulating the amount of polymer and content of HPMC grades with different molecular weights [31].

Gao et al. conducted a mechanistic study of the influence of formulation variables on matrix performance and drug release from HPMC matrix tablets. The effects of HPMC/lactose ratio and HPMC viscosity grade (molecular weight) on solute release and swelling of matrix tablets were investigated. Drug, lactose and HPMC releases were monitored simultaneously. The authors suggested that the strong dependence of HPMC release on viscosity grade can be explained on the basis of the concept of polymer disentanglement concentration. They consider that the HPMC/lactose ratio modulates drug release rate by altering drug diffusivity, while the HPMC viscosity grade impacts matrix dissolution and gel layer thickness development below a critical molecular weight. High-viscosity grade HPMC (>4000 cps), forming slowly dissolving matrices, demonstrates similar drug release rates, mainly due to the same drug diffusivity as a result of the identical gel composition and thickness. The higher apparent drug diffusivity and release rate in fast dissolving matrices (< or = 100 cps) are probably explained by swelling inhomogeneity [32].

The post-hoc test showed that the molecular weight of 750 kDa led to 2.0637 (on average) higher values for t80 compared to 500 kDa, while the highest molecular weight of 1150 kDa only led to 0.3744 higher values for t80 compared to 750 kDa (Table 9).

The obtained results are in accordance with some data published by other authors [23] that the molecular weight of the polymer used is able to influence the release process, but it is significant only within certain limits. In this case, when increasing the molecular weight from 500 kDa (K4M) to 750 kDa (K15M), a difference in the release process was observed, as t80 in similar models increased by an average of about 2.5 h, while when changing the molecular weight from 750 kDa (K15M) to 1150 kDa (K100M), t80 for similar models increased by an average of about 0.32 h.

### 3.5. Influence of the Compression Force—Factor C on t80

The general trend observed among all analogous models in varying the compression force is that as the pressure increases, the release rate slows down, but statistically, the change in this independent variable has the lowest effect on the values of t80. This pattern was more pronounced when comparing the models obtained at 1 t and those at 1.5 t pressure. The differences in the models obtained at 1.5 t and 2 t were insignificant.

As the compression force increases, the density of the obtained matrix system increases, as do the inner interparticle interactions. This leads to a stronger matrix structure and slower water entry in the initial moment, which can probably explain the intensive initial release in the models obtained at the lowest pressure of 1 t.

In a study by Hirun and Kraisit, composite matrix tablets were developed, and the effect of HPMC k-series on porosity, compatibility and release behavior was studied. Scanning electron microscopy was used for the morphological analysis of the top view of the matrix tablets and the side view of the broken tablets. The tablet’s surface morphology was rough, with aggregated particles, small holes and cracks visible in the cross-sections. According to the type of HPMC used, a dense, continuous mass was observed, with some irregularities and rough-breaking pieces. The inner structures of all tablets were composed of solids and air, known as tablet porosity. It can be considered a feature that impacts liquid and drug transport through the solid dosage form. The authors emphasize that the compression force and tablet composition may affect the surface area of the pores and the number of distinct pore diameters in the tablets, thereby affecting the release process [30].

As seen in Table 10, a significant change in t80 was observed when increasing the compression force from 1 t to 1.5 t (0.3137), as well as up to 2 t—0.4548, respectively. A much smaller difference was observed between the two higher compression forces of 1.5 t and 2 t, as the arithmetic mean increase of t80 in this case was only 0.1411. It can be assumed that the increase in the compression force has a significant impact on the release process up to a certain level (in this case, 1.5 t), after which no significant differences are observed. A similar trend has been reported by other authors [33].

**Figure 4:**
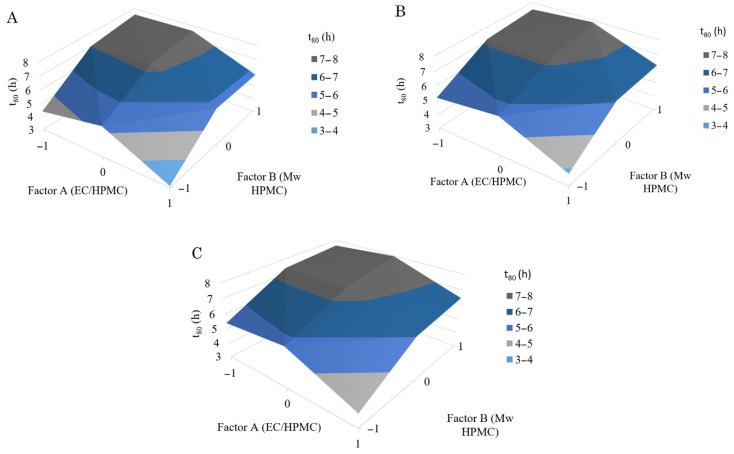
Effect of EC/HPMC ratio and HPMC molecular weight at: (**A**) 1 t compression force (Factor C level 1); (**B**) 1.5 t compression force (Factor C level 0); and (**C**) 2 t compression force (Factor C level 1).

To visualize the correlation between the three parameters, 3D surface plots are presented (Figure 4), explaining the interaction effects of factors A and B at three different levels of factor C.

Table 11 presents statistical tests on the effect of the hydrophobic/hydrophilic polymer ratio (EC/HPMC), the molecular weight of HPMC, the compression force and the correlations between these three factors. The three independent variables used, as well as some of their correlations, influence the process of releasing the included dry plant extract, t80, respectively. The statistical significance of both the individual factors and the partial significance of their combination was established. There are significant differences in the arithmetic mean values for t80 between the different indicators of the independent variables. A significant effect on the release rate of the included dry plant extract was established in the interaction of the hydrophobic/hydrophilic polymer ratio (EC/HPMC) and the molecular weight of HPMC, as well as in the interaction of the molecular weight of HPMC and compression force, while in the interaction of the hydrophobic/hydrophilic polymer ratio (EC/HPMC) and compression force, no statistical significance was found. The value of the coefficient of determination R^2^ indicates what part of the changes in the independent variable will lead to changes in the resultant (dependent) variable, i.e., R^2^ = 0.984 shows that with 98.4% certainty, the changes in the release time of 80% of the included dry plant extract can be explained by the factor variables of the model. The corrected coefficient of determination, R^2^_Adj_., is 0.977. Its value, which is close to 1, indicates that there is a significant linear correlation between the factor variables and the resulting variable. Moreover, the very small difference between the values of R^2^ and R^2^_Adj_. shows that the influence of additional conditions in the statistical model is insignificant.

## 4. Conclusions

The following conclusions can be summarized from the studies conducted on the drug release process and the influence of the studied variables: the concentration of EC significantly affects the release process, and the EC/HPMC ratio of 25/75 is most suitable for prolonging the release process from the formulated polymer matrices and eliminating the initial intensive release. The increase in the molecular weight of the HMPC used led to a delay in the release process, with more significant differences between K4M and K15M (500 kDa and 750 kDa, respectively) and a less pronounced change in the highest molecular weight, K100M (1150 kDa). Although it had a less significant effect on the values of t80, the increase in the applied compression force led to a delay in the release process. F18 can be defined as the most promising model, obtained at a ratio of EC/HPMC 25/75, a molecular weight of HPMC 1150 kDa and a compression force of 2 t, showing the longest time to release 80% of the included active substance (7.97 h). The proposed polymer formulations with dry *Betula pendula* leaf extract can be successfully used as phytotherapeutic products, providing delayed release of the included plant extract. Such pharmaceutical formulations can offer improved biopharmaceutical characteristics, longer maintenance of drug therapeutic plasma concentrations without the need for frequent dosing, reduced risk of abrupt concentration fluctuations and limited drug side effects.

## Figures and Tables

**Table 1:** Composition of the tablet models and production parameters.

Model Code	Extract (g)	EC (g)	HPMC (g)	Talc (g)	Mg-Stearate (g)	Tablet Mass (g)	Compression Force (t)	HPMC Mw (kDa)
F1	0.500	0.072	0.108	0.014	0.006	0.700	1	500
F2	0.500	0.072	0.108	0.014	0.006	0.700	1.5	500
F3	0.500	0.072	0.108	0.014	0.006	0.700	2	500
F4	0.500	0.072	0.108	0.014	0.006	0.700	1	750
F5	0.500	0.072	0.108	0.014	0.006	0.700	1.5	750
F6	0.500	0.072	0.108	0.014	0.006	0.700	2	750
F7	0.500	0.072	0.108	0.014	0.006	0.700	1	1150
F8	0.500	0.072	0.108	0.014	0.006	0.700	1.5	1150
F9	0.500	0.072	0.108	0.014	0.006	0.700	2	1150
F10	0.500	0.045	0.135	0.014	0.006	0.700	1	500
F11	0.500	0.045	0.135	0.014	0.006	0.700	1.5	500
F12	0.500	0.045	0.135	0.014	0.006	0.700	2	500
F13	0.500	0.045	0.135	0.014	0.006	0.700	1	750
F14	0.500	0.045	0.135	0.014	0.006	0.700	1.5	750
F15	0.500	0.045	0.135	0.014	0.006	0.700	2	750
F16	0.500	0.045	0.135	0.014	0.006	0.700	1	1150
F17	0.500	0.045	0.135	0.014	0.006	0.700	1.5	1150
F18	0.500	0.045	0.135	0.014	0.006	0.700	2	1150
F19	0.500	0.018	0.162	0.014	0.006	0.700	1	500
F20	0.500	0.018	0.162	0.014	0.006	0.700	1.5	500
F21	0.500	0.018	0.162	0.014	0.006	0.700	2	500
F22	0.500	0.018	0.162	0.014	0.006	0.700	1	750
F23	0.500	0.018	0.162	0.014	0.006	0.700	1.5	750
F24	0.500	0.018	0.162	0.014	0.006	0.700	2	750
F25	0.500	0.018	0.162	0.014	0.006	0.700	1	1150
F26	0.500	0.018	0.162	0.014	0.006	0.700	1.5	1150
F27	0.500	0.018	0.162	0.014	0.006	0.700	2	1150

**Table 2:** Levels of variation of the independent variables.

Independent Variable/Level	+1	0	−1
EC/HPMC (%)	40/60	25/75	10/90
Molecular weight HPMC (kDa)	1150	750	500
Compression force (t)	2	1.5	1

**Table 3:** Tablet models and levels of coding in the 3^3^ full factorial design.

B	C	A (EC/HPMC Ratio)					
Molecular Weight	Compression Force	Model Code	Level (+1)	Model Code	Level (0)	Model Code	Level (−1)
−1	−1	F1	+1, −1, −1	F10	0, −1, −1	F19	−1, −1, −1
−1	0	F2	+1, −1, 0	F11	0, −1, 0	F20	−1, −1, 0
−1	+1	F3	+1, −1, +1	F12	0, −1, +1	F21	−1, −1, +1
0	−1	F4	+1, 0, −1	F13	0, 0, −1	F22	−1, 0, −1
0	0	F5	+1, 0, 0	F14	0, 0, 0	F23	−1, 0, 0
0	+1	F6	+1, 0, +1	F15	0, 0, +1	F24	−1, 0, +1
+1	−1	F7	+1, +1, −1	F16	0, +1, −1	F25	−1, +1, −1
+1	0	F8	+1, +1, 0	F17	0, +1, 0	F26	−1, +1, 0
+1	+1	F9	+1, +1, +1	F18	0, +1, +1	F27	−1, +1, +1

**Table 4:** Rheological characteristics of granular models.

Granular Models	Tablet Models	Bulk Density, ρ_0_, g/cm^3^ (± SD)	Tapped Density, ρ_s_, g/cm^3^ (± SD)	Hausner Ratio (± SD)	Carr index, % (± SD)	Angle of Repose, ° (± SD)
G1	F1, F2, F3	0.67 ± 0.01	0.78 ± 0.02	1.16 ± 0.07	14.10 ± 0.05	23.54 ± 0.01
G2	F4, F5, F6	0.67 ± 0.03	0.79 ± 0.03	1.18 ± 0.05	15.19 ± 0.07	24.53 ± 0.40
G3	F7, F8, F9	0.66 ± 0.11	0.76 ± 0.02	1.15 ± 0.09	13.16 ± 0.08	23.82 ± 0.04
G4	F10, F11, F12	0.64 ± 0.05	0.76 ± 0.02	1.19 ± 0.08	15.79 ± 0.09	23.40 ± 0.03
G5	F13, F14, F15	0.63 ± 0.05	0.77 ± 0.05	1.22 ± 0.06	18.18 ± 0.07	25.68 ± 0.02
G6	F16, F17, F18	0.65 ± 0.03	0.77 ± 0.06	1.19 ± 0.07	15.58 ± 0.10	22.96 ± 0.02
G7	F19, F20, F21	0.65 ± 0.04	0.78 ± 0.09	1.20 ± 0.12	16.67 ± 0.14	24.56 ± 0.06
G8	F22, F23, F24	0.63 ± 0.04	0.77 ± 0.04	1.22 ± 0.09	18.18 ± 0.09	24.60 ± 0.04
G9	F25, F26, F27	0.63 ± 0.05	0.76 ± 0.09	1.21 ± 0.07	17.11 ± 0.07	24.13 ± 0.05

All measurements were performed three times, and the results are presented as mean ± SD.

**Table 5:** Physicomechanical characteristics of the tablets.

Model	Average Mass, g ± SD	Disintegration Time, min	Friability, % ± SD	Hardness, N ± SD
F1	0.710 ± 0.02	>30	0.126 ± 0.01	88 ± 7.02
F2	0.715 ± 0.04	>30	0.098 ± 0.01	120 ± 9.23
F3	0.709 ± 0.02	>30	0.010 ± 0.02	155 ± 8.50
F4	0.710 ± 0.05	>30	0.122 ± 0.01	96 ± 11.60
F5	0.713 ± 0.09	>30	0.014 ± 0.01	122 ± 5.69
F6	0.709 ± 0.02	>30	0.010 ± 0.02	158 ± 5.80
F7	0.706 ± 0.03	>30	0.090 ± 0.02	106 ± 9.58
F8	0.708 ± 0.04	>30	0.092 ± 0.02	132 ± 10.02
F9	0.705 ± 0.02	>30	0.012 ± 0.03	160 ± 12.67
F10	0.710 ± 0.08	>30	0.110 ± 0.03	95 ± 8.41
F11	0.712 ± 0.05	>30	0.082 ± 0.01	127 ± 6.56
F12	0.710 ± 0.05	>30	0.070 ± 0.01	151 ± 9.63
F13	0.721 ± 0.10	>30	0.116 ± 0.01	96 ± 11.20
F14	0.719 ± 0.11	>30	0.088 ± 0.01	123 ± 10.48
F15	0.723 ± 0.07	>30	0.070 ± 0.01	155 ± 13.40
F16	0.715 ± 0.08	>30	0.084 ± 0.01	102 ± 9.65
F17	0.715 ± 0.08	>30	0.090 ± 0.01	142 ± 7.85
F18	0.712 ± 0.06	>30	0.065 ± 0.01	162 ± 10.14
F19	0.710 ± 0.03	>30	0.115 ± 0.01	92 ± 9.22
F20	0.714 ± 0.04	>30	0.088 ± 0.02	119 ± 11.54
F21	0.711 ± 0.07	>30	0.090 ± 0.02	145 ± 7.25
F22	0.718 ± 0.07	>30	0.100 ± 0.01	100 ± 10.80
F23	0.717 ± 0.10	>30	0.096 ± 0.02	117 ± 14.59
F24	0.717 ± 0.12	>30	0.096 ± 0.03	156 ± 12.52
F25	0.716 ± 0.09	>30	0.100 ± 0.01	98 ± 9.81
F26	0.714 ± 0.11	>30	0.098 ± 0.01	124 ± 9.87
F27	0.715 ± 0.06	>30	0.071 ± 0.01	165 ± 11.74

All measurements were performed three times, and the results are presented as mean ± SD.

**Table 6:** Coefficient of determination (R^2^) and diffusion exponent (*n*).

Model	Zero-Order	First-Order	Higuchi	Korsmeyer–Peppas	Diffusion Exponent, *n*
F1	0.967	0.988	0.999	0.999	0.418
F2	0.978	0.990	0.997	0.999	0.455
F3	0.974	0.993	0.997	0.998	0.528
F4	0.982	0.980	0.997	0.997	0.707
F5	0.982	0.988	0.997	0.998	0.641
F6	0.981	0.984	0.997	0.998	0.653
F7	0.988	0.971	0.993	0.998	0.726
F8	0.995	0.959	0.986	0.999	0.906
F9	0.977	0.960	0.979	0.998	0.947
F10	0.937	0.991	0.980	0.999	0.512
F11	0.949	0.988	0.985	0.992	0.506
F12	0.957	0.993	0.991	0.995	0.477
F13	0.990	0.984	0.996	0.997	0.567
F14	0.988	0.987	0.998	0.999	0.555
F15	0.986	0.987	0.994	0.997	0.571
F16	0.996	0.955	0.992	0.997	0.576
F17	0.995	0.957	0.991	0.997	0.596
F18	0.997	0.968	0.991	0.998	0.657
F19	0.955	0.997	0.991	0.998	0.273
F20	0.974	0.997	0.998	0.999	0.321
F21	0.970	0.998	0.997	0.999	0.354
F22	0.979	0.994	0.998	0.998	0.460
F23	0.984	0.992	0.997	0.998	0.487
F24	0.985	0.991	0.996	0.997	0.513
F25	0.997	0.949	0.989	0.998	0.716
F26	0.997	0.956	0.991	0.999	0.732
F27	0.998	0.952	0.989	0.999	0.744

**Table 7:** t80 values for tablet models F1–F27.

Model	t 80 (h)	Model	t80 (h)	Model	t 80 (h)
F1	3.12	F10	5.26	F19	4.40
F2	3.84	F11	5.71	F20	5.18
F3	3.96	F12	5.78	F21	5.39
F4	5.81	F13	7.25	F22	7.01
F5	6.08	F14	7.12	F23	7.17
F6	6.02	F15	7.38	F24	7.38
F7	5.84	F16	7.63	F25	7.52
F8	6.23	F17	7.86	F26	7.48
F9	6.45	F18	7.97	F27	7.60

**Table 8:** Tukey’s test—hydrophobic/hydrophilic polymer (EC/HPMC).

(I) Hydrophobic/Hydrophilic Polymer Ratio	(J) Hydrophobic/Hydrophilic Polymer Ratio	Mean Difference (I–J)	Std. Error	Sig.
10%/90%	25%/75%	−0.3148	0.05526	0.000
	40%/60%	1.3085	0.05526	0.000
25%/75%	10%/90%	0.3148	0.05526	0.000
	40%/60%	1.6233	0.05526	0.000
40%/60%	10%/90%	−1.3085	0.05526	0.000
	25%/75%	−1.6233	0.05526	0.000

**Table 9:** Tukey’s test—molecular weight.

(I) Molecular Weight HPMC	(J) Molecular Weight HPMC	Mean Difference (I–J)	Std. Error	Sig.
500 kDa	750 kDa	−2.0637	0.05526	0.000
	1150 kDa	−2.4381	0.05526	0.000
750 kDa	500 kDa	2.0637	0.05526	0.000
	1150 kDa	−0.3744	0.05526	0.000
1150 kDa	500 kDa	2.4381	0.05526	0.000
	750 kDa	0.3744	0.05526	0.000

**Table 10:** Tukey’s test—compression force.

(I) Compression Force	(J) Compression Force	Mean Difference (I–J)	Std. Error	Sig.
1 t	1.5 t	−0.3137	0.05526	0.000
	2 t	−0.4548	0.05526	0.000
1.5 t	1 t	0.3137	0.05526	0.000
	2 t	−0.1411	0.05526	0.036
2 t	1 t	0.4548	0.05526	0.000
	1.5 t	0.1411	0.05526	0.036

**Table 11:** Tests of correlations between the technological parameters.

Source	Type III Sum of Squares	df	Mean Square	F	Sig.	Partial Eta Squared (η^2^)
Corrected model	138.768 ^a^	26	5.337	129.448	0.000	0.984
Intercept	3152.323	1	3152.323	76,455.429	0.000	0.999
EC/HPMC	40.019	2	20.009	485.302	0.000	0.947
Molecular weight	93.093	2	46.546	1128.922	0.000	0.977
Compression force	2.927	2	1.463	35.491	0.000	0.568
EC/HPMC × molecular weight	1.122	4	0.280	6.802	0.000	0.335
EC/HPMC × compression force	0.207	4	0.052	1.254	0.300	0.085
Molecular weight × compression force	1.009	4	0.252	6.116	0.000	0.312
EC/HPMC × molecular weight × compression force	0.393	8	0.049	1.190	0.322	0.150
Error	2.226	54	0.041			
Total	3293.318	81				
Corrected total	140.995	80				

^a^ R^2^ = 0.984, R^2^_Adj_. = 0.977.

## Data Availability

The data presented in this study are available on request from the corresponding author.
